# Assessing the perceived awareness and knowledge of type 2 diabetes mellitus clients’ attitude towards telerehabilitation in Limpopo province of South Africa

**DOI:** 10.4102/hsag.v30i0.2891

**Published:** 2025-05-30

**Authors:** Maphoko P. Malema, Jose Frantz

**Affiliations:** 1 Department of Sport, Recreation and Exercise Science, Faculty of Community and Health Science, University of the Western Cape, Bellville, South Africa; 2 Research and Innovation, University of the Western Cape, Bellville, South Africa

**Keywords:** awareness, healthcare, knowledge, telerehabilitation, clients diagnosed with type 2 diabetes

## Abstract

**Background:**

Telerehabilitation is recognised as an effective method for providing rehabilitation services to people who may live in an area where few in-person services are available or those who have mobility or transport issues which make attending rehabilitation sessions difficult or impossible.

**Aim:**

To assess the perception, awareness and knowledge of patients diagnosed with type 2 diabetes and their attitude towards telerehabilitation.

**Setting:**

The study took place in the Elias Motsoaledi Local Municipality.

**Methods:**

The current study followed a quantitative approach using a cross-sectional design to achieve the objectives of the study. A convenience sampling method was used to recruit 202 participants aged 18 years and older. The research data were captured by a double-entry system into a Microsoft Office Excel spreadsheet and were cleared of errors. Thereafter, the data were transferred to the Statistical Package for the Social Sciences version 2024. The data were checked for normality using the Kolmogorov–Smirnov test and was determined to be not normally distributed.

**Results:**

Participants were proportionally represented in terms of age and gender. The majority of participants were aged 56 years and older, which is significantly represented by participants in this study. The current study found that there was no significant awareness, knowledge, attitudes or computer skills regarding telerehabilitation among the participants.

**Conclusion:**

The results highlight the need for educational programmes about telerehabilitation as an alternative method for healthcare services.

**Contribution:**

The study contributes towards assisting in developing an understanding of the awareness, knowledge, attitude and computer skills of patients diagnosed with type 2 diabetes.

## Introduction

The concept of telerehabilitation is central to the present century, where artificial intelligence (AI) is prevalent. Telerehabilitation in Africa is fast becoming a key instrument in the primary healthcare (PHC) sector. Telerehabilitation can provide access to rehabilitation services for people who may live in an area where there are challenges such as mobility or transport issues, making attending rehabilitation sessions difficult or impossible. Telerehabilitation refers to the use of information technology (IT) to aid, assess and provide information to physically or cognitively impaired individuals (Schwamm et al. 2009). Telerehabilitation can provide patients with easy access to rehabilitation services (Cottrell et al. 2017). Telerehabilitation offers and promotes services through information and communication technologies (ICT) to patients in their homes and other environments (Brennan, Mawson & Brownsell 2009). Telerehabilitation empowers and enables individuals to take control of the management of their medical needs and interventions by enabling personalised care, choice and personal control (Brennan et al. 2009).

Telerehabilitation has a number of other benefits, including saving time and costs from the perspective of the healthcare system and the patient (Delaplain 1993), the progression of remote care services for the patient (Russell 2007), the potential effect that reduced travelling might have on the environment, as well as the positive effects of rehabilitating patients in the comfort of their own homes (Russell 2003). Therefore, the quality of life for patients living with non-communicable diseases (NCDs) can be improved by implementing a telerehabilitation program (Nyika 2013). The current study provides an exciting opportunity to advance knowledge of telerehabilitation and could raise awareness of the feasibility of further exploration and implementation. This research contributes to the understanding of how telerehabilitation can be used to complement the existing healthcare inventions in South Africa. It is therefore necessary to explore how telerehabilitation has been received and advanced globally.

One of the first countries that adopted the use of telerehabilitation was the United States of America, with various programmes and initiatives in place for conditions like stroke, traumatic brain injury, Parkinson’s disease and musculoskeletal disorders (Tuckson, Edmunds & Hodgkins 2017). In Canada, telerehabilitation has been used for musculoskeletal disorders, neurological conditions and chronic diseases, as well as in remote and underserved communities (Kairy et al. 2009). European countries, including the United Kingdom, the Netherlands, Denmark and Italy, have implemented telerehabilitation programmes, particularly for stroke rehabilitation, cardiac rehabilitation and pulmonary rehabilitation (Rogante et al. 2010a; 2010b). A review by Cottrell et al. (2017) highlighted the increasing use of telerehabilitation in Europe, with cases focusing on domains such as musculoskeletal, neurological and cardiovascular conditions. A study by Chen et al. (2017a; 2017b) and Choi et al. (2022) reported that their telerehabilitation was explored specifically focusing on rehabilitation for stroke, physical therapy and speech therapy in countries such as China, India and South Korea. These countries have been actively exploring and implementing telerehabilitation services, especially in rural and remote areas, to overcome geographical barriers and improve access to rehabilitation services (Khoja et al. 2016). Countries such as Brazil and Colombia have implemented telerehabilitation programmes, particularly for neurological conditions like stroke and traumatic brain injury (Sarfo et al. 2018; Tozzi et al. 2022).

Telerehabilitation is being regarded as an alternate complementary remote health service. There has been groundwork done to explore the feasibility and efficacy in high-income countries (HICs) (Chen et al. 2017a; 2017b; Wolf et al. 2015) and in some low- and middle-income countries (LMICs) (Chaiyawat & Kulkantrakorn 2012). Therefore, there is a need to establish policies and guidelines in sub-Saharan Africa to facilitate the adoption of telerehabilitation as a complementary healthcare service (Sarfo et al. 2017). Hwang et al. (2017) highlight that there has been minimal progress towards implementing telerehabilitation from patients who have received these services to enable the formulation of a model based on their needs and preferences. Norouzi Aval et al. (2024) conducted a systematic review to examine attitudes, knowledge and awareness of telerehabilitation among health professionals. The results of their review reported that health professionals have positive attitudes towards telerehabilitation.

The present study fills a gap in the literature by attempting to lay a foundation for Southern African studies relating to telerehabilitation. Leochiro et al. (2022) conducted a study about perceptions and experiences about telerehabilitation amongst physiatrists during coronavirus disease 2019 (COVID-19) in the Phillippines. The main results of the study reported that respondents did not have adequate knowledge about telerehabilitation. Even though the implementation of telerehabilitation was successful in Asian countries, there were challenges that were identified, such as infrastructure limitations and the need for cultural adaptation (Gudi, Panakala & Kozhukorova 2022). Sarfo et al. (2017) and Olaleye, Hamzat and Akinrinsade (2017) reported the costs associated with travelling to healthcare facilities for physiotherapy sessions as a challenge. Additional challenges include technological usability issues (Hoaas et al. 2016; Tsai et al. 2016). There is often a lack and unavailability of medical personnel such as physiotherapists, medical doctors, nurses, occupational therapists, etc., which may lead to the deterioration of their health (Sarfo et al. 2018).

The healthcare facilities in the rural areas of Limpopo are very limited, in addition to a shortage of emergency transportation to these healthcare facilities (Gaede & Versteeg 2011). There is a tremendous need for resources in the clinic of Elias Motsoaledi Local Municipality. For telerehabilitation to be successfully implemented, the Sekhukhune district government needs to be involved in improving ICT infrastructure (Ruxwana, Herselman & Conradie 2010). The present study expands on the notion to comprehensively develop experiences and perceptions of telerehabilitation (Hwang et al. 2017). Therefore, the aim of the study was to assess the perceived knowledge, awareness and attitude of patients diagnosed with type 2 Diabetes Mellitus (T2DM) about telerehabilitation. The objectives of this study were: (1) To determine perceived knowledge about telerehabilitation, (2) to investigate the perceived awareness of telerehabilitation and (3) to assess the perceived attitude and computer skills related to telerehabilitation.

## Research methods and design

### Research design

A quantitative research approach was used, following a descriptive cross-sectional design. According to Creswell & Creswell (2017), survey studies help researchers generalise their findings from a sample to a population, allowing inferences to be made about their characteristics, attitudes or behaviour within a specific population (Creswell & Creswell 2017).

### Study setting

The study was conducted in the Elias Motsoaledi Local Municipality on four local PHC facilities and one district hospital in Limpopo province, South Africa. The Elias Motsoaledi Local Municipality (previously called the Greater Groblersdal Local Municipality) is located in the Sekhukhune District of the Limpopo province. According to Statistics South Africa (2011, 2022), over 57% of the population comprises people who speak Northern Sotho, also known as Sepedi.

### Population size and sampling

Only clients diagnosed with T2DM who resided in the Elias Motsoaledi Local Municipality, Sekhukhune district, were recruited. Participants were identified and recruited in liaison with the operational manager or personnel designated by the manager in PHC facilities and the district hospital. A convenient sampling method was used to recruit 202 participants aged 18 years and older. There were 79 male and 123 female participants in this study; there were 116 participants aged 56 years and older; a further 33 participants aged between 36 and 45 years; there were 38 participants aged between 36 and 45 years; 13 participants were aged between 26 and 35 years and lastly, 2 participants were aged between 18 and 25 years. There were 20 participants recruited from the district hospital; a further 50 participants were recruited from PHC 1; 72 participants were recruited from PHC 2; an additional 50 participants were recruited from PHC 3 and lastly, 10 participants were recruited from PHC 4. This method was preferred because it allowed the researchers to recruit participants who met the specific characteristics outlined in the inclusion and exclusion criteria (Golzar, Noor & Tajik 2022). The study’s sample size was calculated using the Slovin formula [n = N ÷ (1+Ne^2^)] (Tejada & Punzalan 2012).

### Inclusion and exclusion criteria

Only clients diagnosed with T2DM were recruited as participants for the current study. Participants were required to comprehend information independently and to understand and speak Sepedi and English. Participants aged 18 years and older were recruited for this study. Participants had to be attending the PHC facilities as outpatients. Participants were excluded if they did not meet the criteria set out in this study.

### Pilot study

For the pilot study, 20 participants with the same eligibility as the official participants but who were not part of the study were recruited through convenient sampling. A test-retest approach was used to check the reliability of the research instrument. This was used to check whether the question content matched the study’s objective, which aids validity (Creswell & Poth 2016).

### Data collection

Data were collected through a self-administered questionnaire. Data were collected over 3 months in four PHCs and one district hospital in the Sekhukhune district. The researchers distributed the questionnaire to clients diagnosed with T2DM. The questionnaire was in English and Sepedi; the researchers was available to interpret and clarify questions that arose. The translated version in Sepedi was prepared by a linguistic specialist and was piloted for clarity. To gather information for the present study, the researchers adopted a questionnaire previously developed and used by Zayapragassarazan and Kumar (2016). The questionnaire was previously used in a study that included 120 health professionals from eight medical colleges in the Puducherry region of India (Zayapragassarazan & Kumar 2016). Participants were able to complete the questionnaire in 15–20 min. The questionnaire included the following subsections: Section A: Demographic information; Section B: Awareness of telerehabilitation. This subsection consisted of 12 items on telerehabilitation, which were used to assess awareness. Each item was assessed with a 3-point Likert scale with scores of 0–2 (0 = don’t know, 1 = heard of it, 2 = know about it). Section C: Knowledge about telerehabilitation. This subsection consisted of 11 items that assessed the level of telerehabilitation knowledge. Each item was assessed with a 2-point scale with scores of 1 (no) and 2 (yes); Section D: Attitude towards telerehabilitation. This subsection consisted of 11 items used to determine the attitude of the respondents towards telerehabilitation. Each item was assessed with a 5-point Likert scale with scores ranging from 0 to 4 (0 = strongly disagree, 1 = disagree, 2 = undecided, 3 = agree, 4 = strongly agree); Section E: Computer skills. This subsection consisted of 13 items to determine the level of awareness of IT and computer skills. Each item was assessed with a 4-point scale with scores of 0–3 (0 = unskilled, 1 = learner, 2 = mediocre, 3 = expert).

### Validity and reliability

Reliability refers to the consistency of the scores from a research instrument (Creswell & Poth 2016). To ensure reliability, the researchers administered the research instrument as a pilot test (Tavakol & Dennick 2011). Therefore, a Cronbach alpha test was used to check the reliability of the research instrument. The overall Cronbach alpha score of (0.87).

### Data analysis

Data were analysed in line with the objectives of the study to assess knowledge, awareness, attitude and computer skills related to telerehabilitation. The data were captured by a double-entry system into a Microsoft Office Excel spreadsheet and were cleared of errors by the researcher and a trained research assistant. Thereafter, the data were transferred to the Statistical Package for the Social Sciences (SPSS) IBM Statistics 2024 (SPSS Inc., Chicago, IL, US) with alpha set at *p* < 0.05 level. The data were checked for normality using the Kolmogorov–Smirnov test and were determined to be not normally distributed. The data were analysed using descriptive statistics (means and standard deviations) (Kaur, Stoltzfus & Yellapu 2018). Spearman’s rank correlation and Kendall’s tau were employed to assess the relationships between variables. A *p*-value of less than 0.05 was used to indicate statistical significance (Templeton 2011).

### Ethical considerations

The present study obtained permission from two institutions, one from the University of the Western Cape’s BioMedical Research Ethics Committee (BMREC reference no.: BM22_6_41) and the second one from the Limpopo province Department of Health (LP 2023 04 023). Participants were informed that their participation was purely voluntary and that they could withdraw their participation from this study at any point without prejudice. Participants’ personal information was not captured on the questionnaire to promote anonymity. The researchers emphasised that participation was voluntary, and participants could withdraw at any time without consequence. No physical risks were involved, and minimal emotional discomfort was anticipated. Participants were given the opportunity to ask questions to address any concerns they had about the study. To respect and observe their confidentiality and anonymity, the identities of the participants were not disclosed at any point during the research process or even after.

## Results

The objectives of this study were: (1) to determine perceived knowledge about telerehabilitation, (2) to investigate the perceived awareness about telerehabilitation and (3) To assess the attitude towards telerehabilitation.

There were 79 male and 123 female respondents. The majority of the participants were aged 56 years and older, and they constituted 57.5% of the overall participants. These results give an impression that the older age group is prone to be diagnosed with T2DM. A minority of the participants fell within the age category of 18 years–25 years (Table 1).

**TABLE 1:** Demographic characteristics of participants.

Characteristics	*n*	%	Mean	Standard
**Gender**	**-**	**-**	1.61	0.489
Male	79	39.1	-	-
Female	123	60.9	-	-
**Age (years)**	-	-	4.53	1.282
18–25	2	1.0	-	-
26–35	13	6.4	-	-
36–45	33	16.3	-	-
46–55	38	18.8	-	-
56 >	116	57.5	-	-

The results in the table above show that the participants in this study have a similar range of awareness relating to telerehabilitation. The results from Table 2 report that participants do not have awareness of telerehabilitation. The mean and standard deviation between male and female participants are similar, indicating that there is no significant difference between the two groups. Both male and female participants in the table above lack awareness of telerehabilitation.

**TABLE 2:** Awareness of telerehabilitation

Variables	Male (*n* = 79)		Female (*n* = 123)	
	Mean	s.d.	Mean	s.d.
Telerehabilitation improves interaction between physicians and patients	0.00	0.000	0.00	0.000
Online health information improves patient’s knowledge	0.00	0.000	0.00	0.000
Telerehabilitation reduces healthcare costs	0.00	0.000	0.00	0.000
Telerehabilitation facilitates medical care	0.00	0.000	0.00	0.000
Telerehabilitation reduces multiple diagnoses	0.00	0.000	0.00	0.000
Telerehabilitation enhances the quality of healthcare	0.00	0.000	0.00	0.000

s.d., standard deviation.

Table 3 reports the participants’ knowledge about telerehabilitation. Neither male nor female participants have knowledge of telerehabilitation. Based on the results above, there is no significant difference in knowledge between male and female participants regarding telerehabilitation.

**TABLE 3:** Knowledge of telerehabilitation.

Variables	Male (*n* = 79)		Female (*n* = 123)	
	Mean	s.d.	Mean	s.d.
Healthcare through the Internet	0.13	0.490	0.37	0.772
Use of the Internet and other electronic technologies to enhance health	0.13	0.490	0.33	0.732
Patient management with drugs through the Internet	0.05*	0.221	0.15	0.385
Patients’ examination conducted through the Internet	0.15	0.483	0.56	0.831
Management of patients including surgical procedures through the Internet	0.08	0.350	0.15	0.444
Electronic medical records of patients’ medical registration and consultations with doctors	0.04*	0.250	0.33	0.707
Follow-up of patients through the Internet or electronic technologies	0.4	0.250	0.42	0.800
Education of physicians through online sources	0.11	0.453	0.47	0.813
Health information exchange and communication in a standardised way	0.04*	0.250	0.49	0.843
Consultation using a video conferencing conversation	0.05*	0.316	0.50	0.853
Association of telerehabilitation	0.37	0.624	0.86	1.027
Communication techniques used in telerehabilitation	2.24	0.895	1.62	0.741
Main source of knowledge	1.11	0.577	1.50	1.035
Main barriers to improving knowledge of telerehabilitation	1.24	0.459	1.39	0.622
Training program attended	0.96	0.192	1.00	0.405
Duration of the programme	0.08	0.385	0.40	0.837

s.d., standard deviation.

* , *p*-value is less than 0.05.

The results illustrated in Table 4 above also report participants’ attitudes towards telerehabilitation. Participants disagreed that telerehabilitation requires effort and threatens information and confidentiality. Furthermore, the participants agreed that telerehabilitation offers a great opportunity; additionally, they believe it is worth using on a trial basis. Therefore, there is no significant difference between male and female participants regarding their attitudes towards telerehabilitation.

**TABLE 4:** Attitude towards telerehabilitation.

Variables	Male (*n* = 79)		Female (*n* = 123)	
	Mean	s.d.	Mean	s.d.
I believe using telerehabilitation requires a lot of mental effort	2.28	0.576	2.60	1.038
In my opinion, telerehabilitation threatens information confidentiality and privacy	1.97	0.225	2.08	0.731
I believe trying the telerehabilitation application is a great opportunity	2.84	0.541	2.60	0.710
I believe using telerehabilitation on a trial basis is enough to see what it could do	3.16	0.373	3.24	0.615
I would like to try the telerehabilitation application before using it	3.18	0.549	3.41	0.746

s.d., standard deviation.

The results presented show that participants are unskilled and can be classified as learners in relation to the computer skills required for telerehabilitation. It is evident from Table 5 that no significant difference was found between male and female participants.

**TABLE 5:** Computer and media skills to use telerehabilitation.

Variables	Male (*n* = 79)		Female (*n* = 123)	
	Mean	s.d.	Mean	s.d.
I have basic information skills, such as the use of a computer and the Internet	0.73	0.473	0.80	0.932
I am able to check IT equipment properly	0.11	0.423	0.14	0.347
I am able to access electronic health records	0.03	0.153	0.03	0.178

s.d., standard deviation; IT, information technology.

## Discussion

The participants in this study were represented in terms of age and gender. The majority of participants were female (*n* = 123) compared to male (*n* = 79). The majority of participants were 56 years and older (*n* = 116), which is significantly represented in this study. Statistics South African (2011, 2022) reported that Sekhukhune District is ranked fourth out of the five municipalities in the Limpopo province. The age group of 55 years and older makes up about 14% of the district’s population, which, based on the results from the current study, does not display an accurate picture.

There are barriers such as lack of awareness, knowledge and attitude related to telerehabilitation that must be overcome before implementing telerehabilitation, especially in rural areas such as the Elias Motsoaledi Local Municipality. Overall, the current study found that there was no awareness, knowledge, attitudes or computer skills regarding telerehabilitation among the participants. There were some significant statements regarding the knowledge about telerehabilitation. The results of this study reported that female participants had a significant perception (0.05*) that telerehabilitation is about patient management with drugs through the Internet. Additionally, female participants had a significant perception (0.04*) that knowledge about telerehabilitation includes electronic medical records of patients’ medical registration and consultation with doctors. Furthermore, there was a significant knowledge about telerehabilitation (0.04*) referring to health information exchange and communication in a standardised way. Furthermore, female participants had a significant (0.05*) perception of knowledge of telerehabilitation to mean direct full consultation of patients through video conferencing.

The results of the current study align with a previous study which reported that awareness, knowledge, attitude and skills related to telemedicine amongst health professionals are inadequate (George, Rozario & Abraham 2007). This corroborates with previous studies on doctors’ knowledge, attitudes and practice regarding telemedicine and e-health in India (George et al. 2007), as well as studies on the knowledge, experience and attitudes of doctors towards telemedicine in the Grampian region (Ruddick-Bracken et al. 2000). Although the study by Ruddick-Bracken et al. (2000) was conducted on health professionals, an argument is made that their lack of awareness and knowledge about telerehabilitation can influence the awareness and knowledge of T2DM patients about telerehabilitation. Therefore, the lack of awareness, knowledge, attitude and skills related to telerehabilitation can be considered a barrier to the implementation of this initiative. A study by Chetty and Mars (2015) explored the potential for telerehabilitation in South Africa, particularly in rural and underserved areas. The study identified challenges such as limited infrastructure, lack of policies and guidelines and the need for training and education (Chetty & Mars 2015).

The results of the present study expand on the findings of Chetty and Mars (2015), particularly considering that awareness, knowledge, attitude and skills are critical for the implementation of any intervention programme. NCDs such as cardiovascular diseases, cancer, chronic respiratory diseases and diabetes mellitus are a significant public health concern in South Africa, accounting for a high burden of disease and mortality (Bradshaw et al. 2018). As such, telerehabilitation services can play an important role in managing and treating many NCDs, helping individuals regain and maintain functional abilities, as well as improving quality of life and preventing complications (World Health Organization 2021). While specific studies on telerehabilitation for NCDs in South Africa are lacking, there is evidence from other countries that telerehabilitation can be effectively used for conditions such as stroke, cardiac rehabilitation, pulmonary rehabilitation and cancer rehabilitation (Cottrell et al. 2017; Rogante et al. 2010a; 2010b; Tonga & Duger 2020). Therefore, telerehabilitation has been proposed as a potential solution to improve access to these services for individuals with NCDs, particularly those living in remote or resource-limited settings (Chetty & Mars 2015).

Saeed et al. (2024) concede that telerehabilitation can become the optimal service delivery method. Telerehabilitation has been identified as a potential and promising solution to address the challenges of limited access and unequal distribution of rehabilitation services in South Africa, particularly in rural and underserved areas (Mars 2013; Mars & Scott 2016). Several studies have reported that the implementation of telerehabilitation has been slow in LMIC compared to other countries globally because of limited income in most families (Brennan et al. 2010). A similar study done by Pramuka and Van Roosmalen (2009) emphasised that most patients in the rural areas of South Africa cannot access rehabilitation services, whether online or face to face, because both are self-funded. Another discovery made by the researchers in this study was that elderly people encounter many challenges concerning the use of telerehabilitation, including the inability to use mobile devices, poor sight, poor hearing and lack of familiarity with smartphones and/or other electronic devices. Studies done by Batsis et al. (2019) and Kruse et al. (2020) reported that the inability to use smartphones and lack of understanding of technology make it difficult for older people to navigate and use the necessary equipment. According to Seifert and Schlomann (2021) and Tsai et al. (2016), the decline in cognitive and sensory abilities that comes with age is another obstacle that complicates the implementation of telerehabilitation.

### Strengths and limitations

The results highlight the need for educational programmes on telerehabilitation as an alternative method for healthcare services. Patients with T2DM may benefit from educational programmes, especially when complemented by physical activity intervention programmes designed to improve their knowledge. The strength of this study is that the results assisted in developing an understanding of the awareness, knowledge, attitude and computer skills of patients diagnosed with T2DM, enabling them to navigate the telerehabilitation services. This allows the data to be used to develop effective educational programmes for these patients, which could be implemented in healthcare facilities within public and private sectors. This is important for incorporating telerehabilitation as an intervention by government entities to combat the overburdened healthcare facilities. A limitation of this study was that it was conducted within one region in the Elias Motsoaledi Local Municipality, which limited the sample size and possibly the overall generalisability to larger PHC facilities in the Sekhukhune district of Limpopo province.
